# The cognitive and perceptual processes that affect observer performance in lung cancer detection: a scoping review

**DOI:** 10.1002/jmrs.456

**Published:** 2021-02-08

**Authors:** Monica‐Rose Van De Luecht, Warren Michael Reed

**Affiliations:** ^1^ Discipline of Medical Imaging Science Faculty of Medicine and Health Sydney School of Health Sciences The University of Sydney Sydney NSW Australia; ^2^ Medical Imaging Optimisation and Perception Group (MIOPeG) Discipline of Medical Imaging Science Sydney School of Health Sciences Faculty of Medicine and Health The University of Sydney Sydney NSW Australia

**Keywords:** Cancer screening, eye‐tracking, lung neoplasms, radiologic detection, visual perception

## Abstract

**Introduction:**

Early detection of malignant pulmonary nodules through screening has been shown to reduce lung cancer‐related mortality by 20%. However, perceptual and cognitive factors that affect nodule detection are poorly understood. This review examines the cognitive and visual processes of various observers, with a particular focus on radiologists, during lung nodule detection.

**Methods:**

Four databases (Medline, Embase, Scopus and PubMed) were searched to extract studies on eye‐tracking in pulmonary nodule detection. Studies were included if they used eye‐tracking to assess the search and detection of lung nodules in computed tomography or 2D radiographic imaging. Data were charted according to identified themes and synthesised using a thematic narrative approach.

**Results:**

The literature search yielded 25 articles and five themes were discovered: 1 – functional visual field and satisfaction of search, 2 – expert search patterns, 3 – error classification through dwell time, 4 – the impact of the viewing environment and 5 – the effect of prevalence expectation on search. Functional visual field reduced to 2.7° in 3D imaging compared to 5° in 2D radiographs. Although greater visual coverage improved nodule detection, incomplete search was not responsible for missed nodules. Most radiological errors during lung nodule detection were decision‐making errors (30%–45%). Dwell times associated with false‐positive (FP) decisions informed feedback systems to improve diagnosis. Interruptions did not influence diagnostic performance; however, it increased viewing time by 8% and produced a 23.1% search continuation accuracy. Comparative scanning was found to increase the detection of low contrast nodules. Prevalence expectation did not directly affect diagnostic accuracy; however, decision‐making time increased by 2.32 seconds with high prevalence expectations.

**Conclusion:**

Visual and cognitive factors influence pulmonary nodule detection. Insights gained from eye‐tracking can inform advancements in lung screening. Further exploration of eye‐tracking in lung screening, particularly with low‐dose computed tomography (LDCT), will benefit the future of lung cancer screening.

## Graphical abstract

Early detection of malignant pulmonary nodules through screening has been shown to reduce lung cancer‐related mortality by 20%. However, perceptual and cognitive factors that affect nodule detection are poorly understood. This review examines the cognitive and visual processes during lung nodule detection.

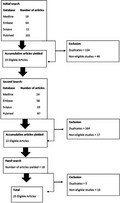

## Introduction

Lung cancer is responsible for the highest mortality rate from cancer worldwide, accounting for 25.3% of all cancer‐related deaths.^1^ The detection of pulmonary nodules, the early indicator of lung cancer through screening has been shown to improve survival from the disease.^2^, ^3^, ^4^ A plain chest X‐ray (CXR) has been the primary imaging tool for suspicion of lung cancer for decades; however, anatomical noise arising from the superimposition of tissues, particularly ribs on nodules in planar images is a major limitation of CXR.^5^ To overcome this limitation of CXR, computed tomography (CT) has been introduced into the screening pathway. Although standard CT imaging has improved the early detection of lung nodules, other conditions such as inflammation, focal fibrosis, pre‐invasive lesions and metastasis can also present with features akin to lung cancer.^6^, ^7^ To optimise CT for lung cancer screening, the identification and follow‐up of nodules ≥ 6 mm with low‐dose CT (LDCT) has been recommended.^7^, ^8^, ^9^, ^10^ Despite a reported 24% reduction in lung cancer mortality due to early detection with LDCT^11^, many countries, including Australia, affirm there remains insufficient evidence to support a national LDCT lung cancer screening programme.^12^ The case against LDCT screening is based on its potential high false‐positive errors and lack of consensus regarding the definition of a positive test and follow‐up strategies. Therefore, the causal factors for these errors and interventions to mitigate them are needed so that the benefits of lung cancer screening can be accrued whilst minimising the associated risks.

Visual search and cognitive processes underpinning image interpretation are inter‐connected and inter‐dependent. Eye‐position analysis using eye‐tracking can provide insight into the often complex and hidden interaction of radiologists with images and can provide information to improve diagnostic performance.^13^ Therefore, understanding the cognitive, visual search, perceptual and decision‐making interactions of observers associated with high diagnostic performance is crucial to inform strategies to optimise the early detection of lung cancer, therefore reducing FP diagnosis, essential for the success of lung cancer screening programmes.

Previous eye‐tracking studies have explored visual search and perceptual behaviours associated with improved pulmonary nodule detection in plain CXR or Chest CT.^13^, ^14^ These studies have reported some significant differences in radiologists’ and radiographers’ behaviours when interacting with images produced by these modalities. Therefore, the visual and cognitive behaviours underpinning lung nodule detection remains unclear and needs to be explored. However, there is a paucity of reviews to provide a better understanding of reader behaviours that impact upon lung cancer detection. Importantly, the differences in visual search and cognitive behaviours of observers between CXR and CT are often poorly understood. Therefore, the purpose of this paper was to review the literature on the visual and cognitive processes associated with the detection of lung nodules in CXR and CT to investigate the similarities and differences in observer behaviour when accurately interpreting plain CXR (two‐dimensional) and Chest CT (three‐dimensional) radiological images.

## Methodology

### Protocol

The Preferred Reporting Items for Systematic Reviews and Meta‐Analyses ‐ Scoping review extension (PRISMA‐ScR) checklist was used for this review.

### Eligibility criteria

Articles were eligible for inclusion if they investigated lung nodule detection in CT or CXR, used native or simulated nodules, involved radiologists (expert viewers), radiology registrars, non‐reporting observers (including radiographers and medical students) and naïve observers were blinded or non‐blinded, utilised calibrated eye‐tracking devices and written in the English Language. Studies conducted between 1978 and 2018 were included in this review as this was the time range of all available studies covering nodule detection during the conduction of this review. Studies that did not fulfil these criteria were excluded as they were conference papers, abstracts, opinion pieces, letters to the editor and comments.

### Information sources

Four databases (Medline, Embase, Scopus and PubMed) were used to search for eligible articles. A Google cross‐search and a hand search using reference lists of published articles were also conducted. Missing data were obtained through contact with the original researchers.

### Search strategy

Search was conducted using a combination of the following search terms: ‘Eye movements’; ‘Eye tracking’; ‘Visual search’; ‘Computed tomography: ‘Lung Nodule’; ‘Lung Cancer’ ‘visual perception’; ‘Gaze characteristics’; lung cancer screening; search terms were combined with either ‘OR’ or ‘AND’.

### Study selection

Studies were assessed for eligibility by applying the inclusion and exclusion criteria to eligible studies. A second reviewer independently confirmed articles for eligibility and disagreements were resolved via discussion.

### Data charting process

A charting table was pre‐drafted, and two authors charted the data together. Data charted included key characteristics and findings such as study design, study population, modality, gaze metric measure(s), nodule size, number of nodules, nodule type and participant characteristics.

### Data items

Variables within data sources included the participants, nodule type, nodule size, lung image source, eye‐tracking model and eye‐tracking analysis measures. Results between studies were compared with the assumption that the variance in image quality and nodule conspicuity was similar although may well have varied especially chronologically between studies due to changing imaging technology.

### Study designs

Only experimental studies were reviewed and this included observer performer studies, cohort studies and randomised control trials. Any eye‐tracking device (head mounted or monitor mounted) was permitted if calibrated. The source of images was considered and recorded in our analysis.

### Types of participants

Studies with participants of various experience in radiology including expert viewers (radiologists), non‐reporting readers (including radiographers and medical students) and naïve observers, were eligible for inclusion.

### Types of outcome measures

Outcomes assessed were functional visual field, gaze volume (total volume of the image that was searched), time to first fixation, time to first pursuit of a nodule, total dwell time (total time a region was looked at), saccade count and visit counts (times an area was gazed upon). Time to first fixation is the time taken for an observer to first gaze upon a region while time to first pursuit is defined as the time to first pursuit is defined as the time taken for an observer to first maintain gaze within a region of interest for greater than 100 msec. Features of sensitivity and specificity were also recorded; false negatives (FN) are findings regarded as missed pathological regions while true negatives (TNs) are regions correctly identified as non‐pathological, false positives (FPs) are regarded as regions incorrectly identified as suspicious while true positives (TPs) are regarded as regions correctly identified as suspicious).

### Data synthesis

Data were synthesised through a thematic narrative approach. Studies were charted in a combined manner and critical appraisals were grounded on methodological rigour and participant experience.

## Results

**Figure 1:**
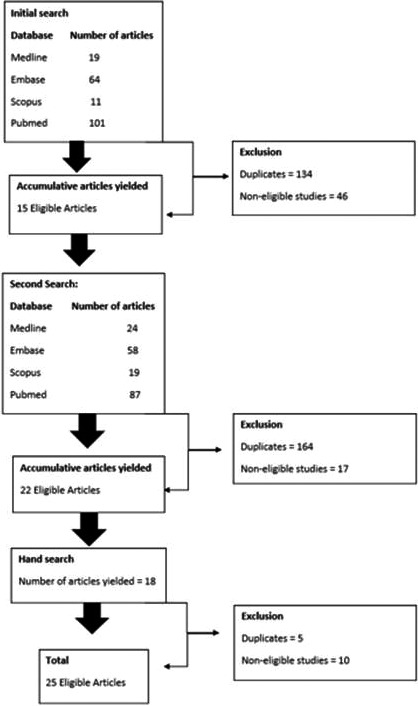
Flow diagram demonstrating sources of data.

The database search resulted in 113 potential articles, and 22 eligible articles were identified. A hand search through the references of published articles resulted in an additional 18 articles of which three were deemed eligible for inclusion (Fig. 1).

Table 1 shows the characteristics of the 25 studies reviewed. The readers’ years of experience varied from 1 to 47 years (mean: 14 years), and 17 were based on CXR and eight on CT. For CXR studies, JAFROC and AFROC figure of merit (FoM) scores ranged from 0.40–0.82.^15^, ^16^ The ROC area under the curve (AUC) scores ranged from 0.85 to 0.93.^17^, ^18^ Sensitivity values ranged from 29 to 90%.^19^, ^20^ For CT studies JAFROC or ROC measurements were not recorded. However, recorded CT sensitivity scores ranged from 12 to 74%.^21^, ^22^, ^23^, ^24^, ^25^, ^26^

**Table 1:** Characteristics of the studies included in the review.

Author(s)	Study design	Eye‐tracker type	Threshold visual angle	Fixation Threshold	Gaze metric	Theme	Number of Readers	Experience (years)	Nodule sizes	Sensitivity
X‐ray studies										
Kundel et al., 1978^12^	OPS	Narco Biosystems			Fixation	Error classification	2	10	1 cm	0.85
Carmody et al., 1981^35^	OPS	Narco Biosystems	1 degree		Dwell Times	Viewing environment	7	NA	1.3 cm	0.95
Nodine & Kundel, 1987^34^	Cohort	Eye track model 200	1 degree	100 ms	Pattern of Fixations	Error classification	2	NA	1 cm	
Kundel et al., 1987^18^	Cohort	Eye track Model 200			Patterns of Fixation, FOV	Satisfaction of search	2	NA	1 cm	0.70–0.90
Kundel et al., 1989^37^	Cohort	Limbus reflection			Visual dwell	Error classification	26	NA		0.75–0.90
Kundel et al., 1990^14^	OPS	Limbus reflection			Dwell times	Error classification	3	NA		0.55
Kundel et al., 1991^19^	OPS	Eye track model 200	1 degree	100 ms	Time to first Fixation	Satisfaction of search	1	10	1 cm	0.40–0.77
Berbaum et al., 1998^29^	Cohort	4100H	3 degree		Dwell Times	Satisfaction of search	20	NA		0.29–0.50
Krupinski et al., 2003^31^	Cohort	ASL 4000SU	2.5 degree	100 ms	Dwell times, Fixation	Error classification	3	>10 years	6 mm–20 mm	NA
Manning et al., 2004^32^	OPS	ASL 504	1 degree	100 ms	Patterns of Fixation	Error classification	4	NA	5 mm–20 mm	0.49–0.62
Manning et al., 2006^15^	OPS	ASL 504	1 degree	100 ms	Patterns of Fixation, FOV	Expertise	8	NA	5 mm–20 mm	NA
Manning et al., 2006^33^	OPS	EYENAL	1 degree	100 ms	Dwell, Fixation	Error classification	8	NA	5–20 mm	0.63–0.82
W. M. Reed et al., 2011^17^	OPS	Tobii x50	1 degree	100 ms	Fixation	Prevalence expectations	22	23		NA
van Geel et al., 2017^28^	RCT	SMI RED		100 ms	Fixation	Satisfaction of search		NA		0.68
Littlefair et al., 2017^27^	Cohort	Tobii x50	2 degrees	200 ms	Dwell Times	Prevalence expectations	17	11 to 27	8–26 mm	0.75–1.0
Donovan & Litchfield, 2013^26^	OPS	Tobii X50	2 degrees	100 ms	Time to first hit	Expertise	10	NA	5–30 mm	0.48–0.60
Reed et al., 2014^16^	OPS	TobiiX50			Dwell Times	Prevalence expectations	22	6 to 42		0.41–0.72
CT studies										
Drew et al, 2013^22^	RCT	SR research 1000			Dwell Times	Satisfaction of search	9	NA		0.85–0.93
Rubin et al., 2015^24^	OPS	SMI IViewX RED	2 degrees		FOV	Satisfaction of search	13	1 to 25	4.8–5 mm	0.12–0.55
Diaz et al., 2015^20^	Cohort	Eye link 100			Pattern of Fixations	Expertise	3	7	4–6 mm	0.30–0.70
Wen et al., 2016^35^	OPS	Eyelink 1000			Pattern of Fixations	Error classification	24	NA		0.61–0.69
Drew et al, 2013^22^	Cohort	Eyelink 1000			Pattern of Fixations	Expertise	25	1 to 40	>3 mm	0.85–0.93
Ebner et al., 2017^23^	Cohort	SMI Iview X RED	2 degrees		FOV	Satisfaction of search	6	6 to 12		0.63–0.74
Williams & Drew, 2017^25^	Cohort	Eyelink 1000			Time to first Fixation	Viewing environment		NA	18–26 pixels	0.47–0.74
Machado et al., 2018^30^	OPS	Tobii 4C	2.5 degrees	100 ms	Fixation	Expertise	3	4		0.43–0.50

OPS: observer performance study; RCT: randomised control trial; FOV: field of view; CT: computed tomography; NA: Not applicable.

## Results from Individual Sources of Evidence

**Figure 2:**
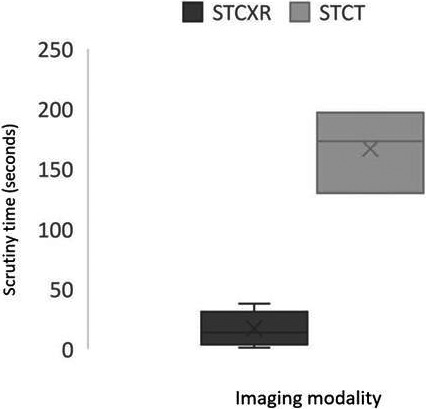
Recorded gaze measures for Computed tomography and Chest X‐ray. STCXR: scrutiny time in chest X‐ray; STCT: scrutiny time computed tomography.

The most commonly charted evidence was dwell time, which refers to the length of visual gaze on a region (typically within a 1° radius) and correlates to different levels of cognitive processing. It is generally assumed that the longer the dwell time the less confident the viewer. Dwell time and time to first fixation were only recorded in CXR studies and ranged from 0.32 to 3.80 seconds^27^, ^28^ and 0.30 and 5.29 seconds,^17^, ^18^, ^28^ respectively. The scrutiny time for CXR and CT are shown in Figure 2.

## Thematic Findings

### Functional visual field and the satisfaction of search

In CXR, a 5° radius was the threshold for the maximum functional field of view, and a visual field beyond 5° contributed little to the discovery of a lesion.^19^, ^20^ Coverage and nodule detection were similar for human observation and random computer scanning (a computer observer that was programmed to scan the radiological image in a random manner) in the first 10 seconds of viewing after which the human observer demonstrated greater coverage but this coverage had no significant effect on nodule detection.

In CT, total field of coverage, varied widely, from 26.7% to 69%.^22^, ^23^, ^25^ When gaze was further than 50 pixels from the nodule, there was less than 1% chance of detecting the nodule.^25^

Systematic viewing (viewing in a structured and ordered manner opposed to randomly) in X‐ray did not improve sensitivity (*P *= 0.30) or specificity (*P *= 0.73) compared to non‐systematic methods.^29^ Systematic training instructs readers to view images in a sequential manner, while non‐systematic training instructs readers to identify suspicious regions while randomly scanning images. Although systematic viewing increased coverage, it also resulted in decreased specificity, 44.7% in systematic training vs. 60.3% in non‐systematic training.^29^ Systematic viewing methods performed similar to random scanning in respects to image coverage and nodule detection in the first 10 seconds of viewing when approximately 80% of nodules are detected.^19^ After 10 seconds, nodule detection plateaued indicating similar performance between random and systematic viewing methods.^19^ Additionally, FN findings with zero dwell were rare, suggesting incomplete satisfaction of search was not responsible for missed lesions.^30^

Search volume (defined as the volume of lung parenchyma within 2°–2.5° of all recorded gaze points) was associated with increased nodule detection, increasing sensitivity from 55% to 91%.^25^ Experienced radiologists covered 17% more of the lung field and made 12% more TP decisions compared to inexperienced readers^22^. These experienced radiologists viewed on average 25 more CT scans a week than their inexperienced counterparts^22^. Experts were less susceptible to inattentional blindness; a phenomena whereby obvious abnormalities are missed because viewer attention was concentrated elsewhere (i.e. in identifying a nodule).^22^, ^23^

### Expertise patterns of search

Expert search patterns were determined based on observer performance and the correlation with viewing patterns. An expert was then defined according to their viewing style. In CXR, experts viewed images in long sweeps with greater average fixation distance.^16^ This was similar in CT viewing where experts demonstrated fewer analysis patterns and fixation clusters.^21^, ^31^ In CT, experts exhibited driller (observers that focus on one region of the lung at a time while scrolling through image stacks) characteristics while novice readers were typically scanners (scanning across one slice of a CT before moving on to the next slice).^22^ Drillers had on average more experience in CT chest viewing of around 45 cases per week compared to scanners who averaged 20 cases per week.^22^ Experts’ had greater distance between fixations and made 5% fewer search errors.^22^, ^27^ In addition, experts had faster viewing times, larger sweeps of visual search^16^ and focused on the lung apices^31^ while naïve and non‐expert viewers searched regions of the lungs with low probability of containing a lung nodule.^27^ Although the viewing style demonstrated by experts is beneficial to the efficient detection of a nodule it does not aid in satisfaction of search and the identification of other comorbidities that could be present in CXRs.

### Classification of errors through dwell time

**Figure 3:**
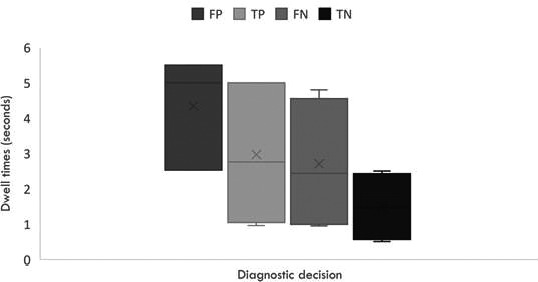
Dwell time for different diagnostic decisions.

The total dwell of TPs, TNs, FPs and FNs in CXR are shown in Figure 3. Time to first hit is defined as the time taken for an observer to first gaze at a region. The time to first hit of FNs was 0.12 seconds longer than for TPs^32^, and 50%‐ 80% of TN decisions (fixations on a lesion‐free area that does not yield a report) are made within the first second of fixation but 80% of all positives occurred with longer visual scrutiny (>3 seconds).^33^, ^34^ In 65% of cases, FNs were fixated on for longer than one second and all missed nodules fixated on had an average dwell time of 3.10 seconds.^33^ In one study, all decisions not to report a nodule made after three seconds were incorrect; however, it must be noted that data from a single study cannot be generalised to radiological practice.^34^ Overall performance improved by 16% when a second opportunity to read the image was aided by visual feedback according to dwell times.^15^, ^35^ Like TPs, FPs are characterised by dense fixation clusters over the region.^16^, ^38^ In CT, scanners were more likely to commit search errors (60%) compared to drillers (30%).^22^, ^36^

### Viewing environment and comparative scanning

In CT, disruptions to search increased search time by 8% and significantly increased refixation rates (*P* = 0.029).^26^ Overall, interruption impaired accurate search resumption.^26^ In CXR, comparative scanning (making visual comparisons between image regions with suspicious perturbations and normal image features in order to discern whether the suspicious region contains an abnormality) increased the total number of low‐confidence TP decisions.^37^

### Prevalence expectations

**Figure 4:**
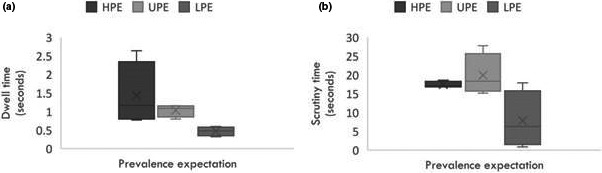
Gaze measures at different prevalence expectations in relation to dwell time (a) and scrutiny time (b).

The effect of prevalence expectation on visual search was assessed in CXR and the gaze measures are shown in Figure 4. Time to first fixation was longer at high (0.48–5.29 seconds) than low (0.33–3.42 seconds) expectation. Prevalence expectation did not impact diagnostic performance. Dwell time was significantly longer for TPs (1.75 seconds) than FNs (0.31 seconds)^17^, ^18^, ^37^ at high expectation and FNs at unknown prevalence expectation (0.48 seconds, *P* = 0.008)^28^.

## Discussion

Reader interaction with radiological images is a broad concept which cannot be defined in a single measure. We identified five main themes related to the pursuit of lung nodules in CXR and CT using eye‐tracking: functional visual field, expertise related patterns of search, classification of errors through dwell time, the viewing environment’s effect on search and the effect of prevalence expectations on visual search. Our findings on functional visual field in CXR and CT varied between these modalities. The 5° useful field of view in CXR was reduced to 2.7° in CT. This difference may be due to the complexity of viewing dynamic CT scans.^24^ For example, in contrast to superimposed image features in 2D images, CT features are only transiently available (‘pop‐out phenomenon’).^24^ This phenomenon mimics motion, stimulating physiologically distinct retinal detection mechanisms and distorts the effects of image contrast and anatomical noise.^24^ A better understanding of this physiological challenge in CT viewing may provide informed strategies for tailoring radiologist training or alternative display paradigms such as incorporating eye‐tracking feedback responsive to the typical signs of error to enhance search.

We observed a strong association between coverage and true positive decisions, suggesting that incorporating search strategies that increase coverage into observers practice may improve diagnostic performance by increasing the number of nodules that fall within the visual field. However, no association was found between systematic viewing and sensitivity or specificity.^29^, ^30^ These findings suggest that, for the sole purpose of pulmonary nodule detection, it may be more important to tailor radiology training around abnormality recognition, with emphasis on coverage of image locations where malignant nodules are more likely to develop rather than systematically searching the whole image. Such a strategy is increasingly important to reduce viewing time, particularly with increasing radiologist workload. However, to avoid satisfaction of search and for the detection of other non‐nodule abnormalities, a more thorough search may be more suited. There were wide variations in image coverage, which may be linked to premature termination of search due to a reader’s confidence that the most pressing abnormality had been identified. The differences in eye‐tracker technologies (see Table 1) may have contributed to these discrepancies. For example, the maximum extent of useful visual field in X‐ray image interpretation has been shown to be 5°;^19^, ^20^ however, there were variations in the calibration of eye‐tracker models from 1° (in newer models) to 3° (in older models) of human gaze (Table 1). This may underestimate peripheral vision and its contribution to global search. While this human limitation in visual search can be mitigated, it is important that the calibration of eye‐tracking devices can be standardised to provide better observer feedback aids to overcome human search limitations and premature search termination.

Although visual search is inherently different between CXR and CT viewing, this review found that expert search was similar between the two modalities: experts focused on regions likely to contain a nodule, analysed large amounts of information with less exhaustive search patterns demonstrating greater distance between fixation points, made fewer saccades and committed at least 5% fewer search errors.^16^, ^22^, ^27^, ^28^ Thus, the ability to select relevant information for further processing distinguishes experienced (qualified radiologists) from inexperienced (radiology residents and non‐reporting radiographers) observers, suggesting the need for interventions that improve the understanding of pathological features and the tailoring of training around abnormality recognition with focus on the areas that are more likely to contain malignancy.

There was variation in time to first fixation between CXR studies. This variation could arise from differences in task difficulty and the expertise of the readers. For example, some studies used naïve viewers without training and qualification in interpreting and diagnosing radiographic images of the chest (e.g. civilians) while others utilised radiologists with 11–23 years of experience post‐certification (Table 1). In addition, simulated nodules appeared more obvious and recorded shorter dwell times. We found that time to first fixation for FNs was significantly longer than that reported for TPs, suggesting that missed nodules fail to attract the same level of visual attention as TPs. However, the total dwell time of FNs and TPs were similar (Fig. 3). It is not clear why, despite these similar dwell times, some lesions are rejected. However, since fixation duration is an indicator of cognitive processing, the reason for FN must lie in the often hidden intricate decision‐making process. It was observed that prompted reconsideration of uncertain areas that attracted prolonged dwell in CXR resulted in more accurate diagnosis,^15^ and supports the need for feedback mechanisms to prompt the readers to reconsider suspicious areas in X‐ray images and could be attempted in CT viewing. Collaborative computer‐aided diagnostics utilising both eye‐gaze metrics and computerised tumour recognition software have been successfully demonstrated in imaging^39^ and should be explored in lung cancer screening to reduce FN errors.

We found that FP decisions received the longest dwell times (Fig. 3). While nodule features contribute to its detectability, no relationship was found between nodule features and visual dwell.^32^ Unfortunately, FP errors are a major limitation of lung cancer screening computer‐aided tools. Therefore, studies are required to establish image features that mimic FPs so that these can be used to inform radiologists’ training and technological innovations to reduce FP errors. TN decisions were the quickest decisions followed by FNs; however, FNs with zero dwell were rare, suggesting incomplete search was not responsible for missed lesions. Compared to CXR, decision errors were less common in CT, reflecting an improvement in radiological features that aid decision‐making. Since the low‐exposures of LDCT increases noise and reduces the visibility of low attenuating nodules, it is possible that the distribution of errors may differ in LDCT. However, the authors are aware of no eye‐tracking study has been conducted on LDCT to date. The sensitivity for CT (12–74%) was generally less than that recorded for CXR (29–90%).^21^, ^22^, ^23^, ^24^, ^25^, ^26^ The authors suspect that this is because for X‐ray studies using sensitivity measures the nodules were larger (1 cm) than the nodules used in the CT studies using sensitivity measures (3–20 mm).^13^, ^29^, ^37^

Interruptions increased search time by 8% and impaired the ability to continue search accurately without impacting diagnostic performance.^26^ Comparative scanning increased the probability of detecting low and medium contrast nodules and comparison of normal lesion‐free areas against suspicious regions improved decision‐making. Lung cancer screening programmes select individuals with high risk of lung cancer. Radiologists, therefore, inherently expect a high prevalence of lung nodules, which anecdotally may influence visual search and cognitive interaction with screening images. We did not find any association between prevalence expectation and diagnostic performance or time to first fixation; however, it increased dwell times and number of total fixation clusters.^17^, ^18^, ^28^ This suggests that with high prevalence expectations, experts feel compelled to examine the image in greater detail.

The limitations of this review include that we could not retrieve missing information from some sources, particularly sources that did not report FP dwell times. Secondly, variations in methodologies made it difficult to compare findings. For example, diagnostic performance was measured through a combination of ROC, JAFROC and sensitivity and specificity measures. Each method varies in the process of quantifying performance and may not be linearly comparable. In addition, differences in task difficulty between studies including radiographic images datasets, nodule characteristics and reader characteristics make it difficult to compare studies. Finally, search was restricted to eye‐tracking studies but qualitative and think‐aloud studies might have also been useful in supplementing insight into cognitive processes associated with lung nodule detection.

In summary, there are differences in functional visual field and decision‐making errors between CXR and CT images when searching for lung nodules. In both modalities, greater visual coverage is associated with higher nodule detection and experts view images in long sweeps with greater average fixation distance. No direct relationship has been shown between factors such as incomplete search, interruptions and prevalence expectation and diagnostic performance. Comparative scanning is associated with increased nodule detection and poor decision‐making accounts for most of the diagnostic errors. Insights gained from this review should inform the development of educational interventions and feedback models to reduce future image interpretation errors in lung cancer detection.
